# Commentary on: Analysis of Nipple-Areola Complex Localization Using Male Cadavers: Considerations for Gender-Affirming Surgery

**DOI:** 10.1093/asjof/ojab037

**Published:** 2021-10-16

**Authors:** Bardia Amirlak

**Affiliations:** associate professor of plastic surgery, Department of Plastic Surgery, UT Southwestern Medical Center, Dallas, TX, USA

Double incision female to male mastectomy, with free nipple areola complex (NAC) grafting is the most common chest masculinization surgery performed.^1^ The authors sought to answer an existing debate regarding the most optimal bony and muscular anatomical landmarks for NAC placement.^2^ They used 25 formaldehyde-embalmed male cadavers and conducted various measurements of the NAC and surrounding structures.

Findings from the authors study support the hypothesis that certain physical factors such as Body Mass Index (BMI) impact the measurements for the NAC in relation to certain anatomical landmarks. For example, the anterior axillary line and medial border of the pectoralis major muscle (PMM) may be less reliable. The authors’ findings corroborate previous studies,^3,4^ that the most natural shape for a male NAC is horizontal oval, with placement falling somewhere between the fourth and fifth intercostal space. There was no correlation found between age, weight, height or BMI, and the intercostal space the NAC was located. The authors concluded that similar to a previous study,^5^ placing the nipple 2.5 cm medial to the lateral border of the pectoralis major muscle and 2.4 cm above its inferior border provides the most consistent measurement for a masculine appearing chest. Although the pectoralis landmarks can be helpful for intraoperative NAC placement, factors such as chest shape and muscular development due to preoperative hormone replacement therapy may play a role in the relationship between NAC and PMM. Therefore, regardless of the authors finding, a consistent location of the NAC in relation to fixed landmarks in transmen needs a more comprehensive analysis, with taking various body types and higher BMI patients into consideration. The cadaver BMIs in this study were not more than 32 and due to the severity of the dysphoria, this surgery is commonly performed in higher BMI patients. In addition, the average cadaver age was 75 years old, which would be a big limitation to this study as the location and shape of the NAC can change with aging skin, fat and muscle wasting or even postmortem changes.^6^ In the Video, we discuss and comments on some of these findings in more detail.

Trans men are rightfully becoming better informed and expect specific masculinizing results. A mal-positioned or distorted areola is difficult to correct. It is important to not only use appropriate landmarks, but also confirm the final position of the NAC based on visual confirmation while patients are sat up in surgery, unbiassed from measurements from the pectoralis major muscle.
